# Integrative Network Toxicology Reveals Potential Molecular Targets Linking Plasticizer Exposure to Inflammatory Gastrointestinal Disorders

**DOI:** 10.3390/genes17060667

**Published:** 2026-06-07

**Authors:** Yongqi Chen, Jiyuan Shi, Yun Ruan, Jinghan Guan, Miaohan Yan, Zongying Zhang, Luojin Wu, Mengmeng Sang, Xinfeng Wang, Liming Mao, Zhaoxiu Liu

**Affiliations:** 1 Department of Gastroenterology and Hepatology, Affiliated Hospital of Nantong University, Medical School of Nantong University, Nantong 226001, China; 2431320326@stmail.ntu.edu.cn (Y.C.); 2231510015@stmail.ntu.edu.cn (Y.R.); 2113310013@stmail.ntu.edu.cn (Z.Z.); 2431310023@stmail.ntu.edu.cn (L.W.); 2 Department of Immunology, School of Medicine, Nantong University, Nantong 226001, China; 2331310021@stmail.ntu.edu.cn (J.S.); 2531110377@stmail.ntu.edu.cn (J.G.); 2531110370@stmail.ntu.edu.cn (M.Y.); sangmm@ntu.edu.cn (M.S.); 3 Basic Medical Research Center, School of Medicine, Nantong University, Nantong 226019, China; 4 Jiangsu Province Key Laboratory in University for Inflammation and Molecular Drug Target, Nantong University, Nantong 226019, China; 5 Department of Hematology, Affiliated Hospital of Nantong University, Medical School of Nantong University, Nantong 226019, China; wxf5204079@126.com

**Keywords:** DMP, DOP, DEP, ATBC, network toxicology, molecular docking, inflammatory gastrointestinal disorders

## Abstract

Background: Plasticizers, including phthalate esters and phthalate-free alternatives, are widely detected environmental chemicals. Although increasing evidence suggests that plasticizers may disrupt gastrointestinal homeostasis, their potential molecular links with inflammatory gastrointestinal disorders (IGDs) remain unclear. Methods: This study aimed to systematically identify potential molecular targets and pathways linking representative plasticizers with IGDs. An integrative network toxicology framework was applied to investigate four plasticizers, including dimethyl phthalate (DMP), diethyl phthalate (DEP), dioctyl phthalate/di(2-ethylhexyl) phthalate (DOP/DEHP), and acetyl tributyl citrate (ATBC), in relation to Crohn’s disease (CD), ulcerative colitis (UC), esophagitis, and gastritis. Plasticizer- and disease-related targets were collected from public databases, followed by overlapping target screening, protein–protein interaction network analysis, functional enrichment analysis, GEO-based transcriptomic validation, molecular docking, molecular dynamics simulation, and single-cell RNA-seq analysis. Results: Disease-specific candidate targets were identified, including *CXCL8* and *FN1* for CD, *IL1B* for UC, *MAPK3*, *FASN*, *FN1*, *PPARG*, *CXCL8*, *FOS*, and *HIF1A* for esophagitis, and *MMP9*, *TNF*, *TLR4*, *IL6*, *CCR2*, *IFNG*, and *PTGS2* for gastritis. Cross-disease analysis further identified plasticizer-associated signature targets, including *MMP7* for DMP, *HMOX1* and *NOS2* for DEP, and *LTF* and *CCL11* for ATBC. Enrichment analysis indicated that these targets were mainly involved in inflammatory, chemokine, MAPK-related, and xenobiotic response pathways. Molecular docking and dynamics simulations suggested stable interactions between selected plasticizers and candidate targets, while single-cell analysis revealed their cell-type-specific expression patterns in epithelial, immune, and stromal compartments. Conclusions: This study provides an exploratory network toxicology framework for identifying potential molecular associations between plasticizer exposure and IGDs. The findings highlight disease-specific and plasticizer-associated candidate targets that may guide future experimental validation and environmental risk assessment.

## 1. Introduction

Plasticizers are chemical additives widely used to improve the flexibility, durability, and processability of plastic materials. Among them, phthalate esters, including dimethyl phthalate (DMP), diethyl phthalate (DEP), and dioctyl phthalate/di(2-ethylhexyl) phthalate (DOP/DEHP), represent some of the most extensively used plasticizers. Acetyl tributyl citrate (ATBC), a non-phthalate plasticizer, has also been increasingly applied as an alternative in food-contact materials, medical products, personal care products, and other consumer goods [1,2]. ATBC is a phthalate-free plasticizer widely used as an alternative to conventional phthalates in food-contact materials, medical devices, toys, and consumer products. ATBC does not belong to the phthalate ester family. Phthalates possess an aromatic benzene ring as the core structure, while ATBC is an aliphatic citrate ester without aromatic rings. Although both contain ester bonds, their parent skeletons, functional groups and chemical classifications are distinctly different. All four compounds are widely used as plasticizers for plastics, so they are often compared in the same application scenarios. Because plasticizers are not covalently bound to polymer matrices, they can migrate from plastic products into food, water, dust, and biological systems, resulting in continuous human exposure through ingestion, inhalation, dermal absorption, and medical contact [3].

Growing evidence suggests that plasticizers and their related compounds may pose substantial risks to human health [4,5]. Phthalates and their metabolites have been associated with endocrine disruption, reproductive toxicity, metabolic abnormalities, immune dysregulation, oxidative stress, and inflammatory injury. The gastrointestinal tract is particularly vulnerable to plasticizer exposure because it represents a major route of chemical intake and a direct interface between environmental toxicants, intestinal epithelial cells, gut microbiota, and mucosal immune responses. Long-term exposure to plasticizers may impair epithelial barrier integrity, alter tight junction function, disturb gut microbiota homeostasis, and activate inflammatory signaling pathways, thereby contributing to chronic mucosal injury and gastrointestinal dysfunction [6].

Inflammatory gastrointestinal disorders (IGDs) are complex diseases driven by interactions among genetic susceptibility, immune dysregulation, environmental exposure, microbial imbalance, and epithelial barrier dysfunction [7]. Epidemiological studies indicate that inflammatory gastrointestinal diseases represent a substantial and increasing global health burden. For example, the global prevalence of ulcerative colitis was estimated to be approximately five million cases in 2023, and the incidence of inflammatory bowel disease has continued to rise in many regions worldwide. GERD-related esophageal inflammatory conditions and gastritis/duodenitis are also highly prevalent upper gastrointestinal disorders and contribute substantially to global digestive disease burden. Crohn’s disease (CD) and ulcerative colitis (UC), the two major forms of inflammatory bowel disease, are characterized by chronic relapsing intestinal inflammation, abdominal pain, diarrhea, and progressive tissue damage [8]. Esophagitis and gastritis are common upper gastrointestinal inflammatory disorders involving epithelial injury, immune-cell infiltration, cytokine activation, and tissue remodeling [9,10,11]. Given that plasticizer exposure can promote oxidative stress, barrier disruption, immune activation, and microbiota disturbance, these compounds may be associated with the initiation or progression of CD, UC, esophagitis, and gastritis.

Several studies have begun to link plasticizer exposure with disease progression. Phthalates have been implicated in inflammatory, metabolic, endocrine, reproductive, and tumor-related disorders, suggesting broad systemic toxicity. In gastrointestinal contexts, experimental studies indicate that phthalate exposure may aggravate intestinal inflammation by disrupting epithelial barrier function, reshaping gut microbiota composition, and enhancing pro-inflammatory mediator production [12]. Network toxicology studies have also suggested that plasticizers may affect disease progression by interacting with key proteins and signaling pathways. However, current evidence remains fragmented, and most studies have focused on individual compounds, isolated toxic endpoints, or single disease models [12,13,14,15,16]. The potential effects of DMP, DEP, DOP/DEHP, and ATBC on CD, UC, esophagitis, and gastritis have not been systematically compared.

Therefore, the molecular mechanisms linking specific plasticizers to distinct IGDs remain insufficiently defined. In particular, it remains unclear whether different plasticizers converge on shared inflammatory pathways or act through disease-specific molecular targets. It is also unknown which candidate targets may mediate plasticizer-associated toxicity across lower and upper gastrointestinal inflammatory diseases.

To address these gaps, the present study established an integrative network toxicology framework to systematically investigate the potential links between four representative plasticizers—DMP, DEP, DOP/DEHP, and ATBC—and four IGDs, including CD, UC, esophagitis, and gastritis. First, putative plasticizer-associated targets and disease-related targets were collected from public databases and integrated to identify overlapping toxicant–disease targets. Protein–protein interaction network analysis, topological screening, and functional enrichment analysis were then performed to prioritize key targets and perturbed biological pathways. These candidates were further refined using transcriptomic differential expression analysis across disease-relevant GEO datasets. Finally, molecular docking and molecular dynamics simulations were applied to evaluate the binding affinity and structural stability between representative plasticizers and prioritized targets. Single-cell RNA-seq analysis was then used to characterize the cell-type-specific expression patterns of key targets in diseased tissues. Through this multi-step strategy, this study aimed to identify disease-specific and plasticizer-associated molecular targets and provide mechanistic insights into how plasticizer-related compounds may contribute to inflammatory gastrointestinal disease progression.

## 2. Materials and Methods

### 2.1. Toxicological Analysis of DMP, DEP, DOP, and ATBC

Toxicity predictions for the compound structural models of DMP, DEP, DOP, and ATBC were conducted using the ADMETlab 3.0 and ProTox-II databases to obtain relevant information regarding their induced toxicity [17,18] (Supplementary File S1). The SMILES sequences for DMP, DEP, DOP, and ATBC were sourced from the PubChem database.

### 2.2. Collection of Targets for DMP, DEP, DOP, and ATBC

For this study, target prediction was conducted through the ChEMBL [19], PubChem, and SwissTargetPrediction [20] databases for target prediction, with the species restricted to *Homo sapiens*. After merging and removing duplicate entries from the predicted results, all unique targets were retained for subsequent analyses. The UniProt standardization process was then applied, using the IDMapping service to convert target gene names from different databases into UniProtKB identifiers. Subsequently, the UniProt IDMapping service was employed again for batch conversion, yielding a standardized and unified Gene Symbol. Finally, a rigorously validated standardized target library was established for subsequent analyses, ensuring the reliability and reproducibility of the research findings.

### 2.3. Selection of the Target Network Related to Digestive System Toxicity

Common digestive diseases were classified into three categories: inflammatory bowel disease (IBD), esophagitis, and gastritis. IBD included CD and UC. The disease terms were input into the GeneCards [21], DrugBank [22], and OMIM [23] databases to identify relevant targets. For disease-related target collection, only targets with a relevance score greater than 50 were retained from the GeneCards database, whereas all targets obtained from the DrugBank and OMIM databases were included in the subsequent analyses. Subsequently, the UniProt standardization process was applied, using the IDMapping service to convert target gene names obtained from two databases into UniProtKB identifiers (selecting Homo sapiens entries). The predicted targets were merged, and duplicates were removed to construct a standardized target database for CD, UC, esophagitis, and gastritis. Next, a Venn diagram was employed to independently identify common targets between the toxic compounds DMP, DEP, DOP, ATBC and CD, UC, esophagitis, and gastritis. The UniProtKB identifiers of the common targets were uniformly converted to gene names (selecting Homo sapiens entries) for subsequent analyses. These intersecting genes were considered potential targets through which two toxic compounds may induce digestive system toxicity.

### 2.4. Construction of Protein Interaction Network and Screening of Hub Targets

The intersection genes identified as being involved in the digestive system toxicity induced by DMP, DEP, DOP, and ATBC were input into the STRING [24] database. The species was restricted to Homo sapiens, and the minimum required interaction score was set to “medium confidence > 0.4” for analysis to obtain the protein–protein interaction (PPI) network diagram. These results were imported into Cytoscape 3.10.4, hub genes were identified using the cytoHubba plugin in Cytoscape with default parameter settings. The central targets of DMP, DEP, DOP, and ATBC were derived from the important nodes obtained by three different topological analysis methods, including degree centrality (DC), betweenness centrality (BC), and closeness centrality (CC) [25]. Typically, the top fifteen ranked nodes from each method were selected for intersection analysis.

### 2.5. Analysis of Target Protein Function and Pathway Enrichment

To elucidate the core mechanisms and pathways associated with the potential targets related to respiratory toxicity induced by DMP, DEP, DOP, and ATBC, Gene Ontology (GO) and Kyoto Encyclopedia of Genes and Genomes (KEGG) pathway enrichment analyses were conducted [26]. Functional annotation and pathway analysis were performed using the DAVID database, with Homo sapiens designated as the reference species. GO analysis covered one-dimensional biological process (BP), to clarify the primary biological functions [27]. KEGG enrichment analysis was used to identify significant pathways related to the potential targets of respiratory toxicity caused by these toxic compounds. Results with statistical significance (adj.*p* < 0.05) were prioritized for analysis, with the top ten enriched KEGG pathways and GO terms subsequently visualized through bioinformatics tools to facilitate interpretation and visualization of the analytical outcomes.

### 2.6. Screening of Key Toxic Targets

To improve the reliability of core toxic target screening, transcriptomic datasets related to digestive inflammatory diseases were downloaded from the GEO database for validation, including GSE24287 [28], GSE59071 [29], and GSE36807 [30] for CD; GSE13367 [31], GSE24287, and GSE179285 [32] for UC; GSE58640 [33], GSE190027 [34], and GSE234973 [35] for esophagitis; and GSE60427 [36], GSE47797 [37], and GSE60662 [38] for gastritis. To reduce differences among datasets, all differential expression results were standardized using gene symbol, log_2_FC, adjusted *p*-value(adj.*p*), and dataset label, and DEGs were screened with the same criteria: adj.*p* < 0.05 and |log_2_FC| ≥ 1. For hub target identification, the top 15 targets ranked by degree centrality, betweenness centrality, and closeness centrality were selected from the PPI networks, and their overlapping targets were retained to reduce the bias of a single topological parameter. These hub targets were then cross-analyzed with DEGs from the corresponding GEO datasets, and targets that were both topologically important and significantly differentially expressed were defined as core toxic targets. Bar–volcano plots were generated using ggplot2 to visualize the expression patterns of these core targets.

### 2.7. Molecular Docking and Toxic Targets of DMP, DEP, DOP and ATBC

Molecular docking was performed to investigate the interactions between DMP, DEP, DOP, ATBC and their corresponding toxic targets. The main software packages employed included PyMOL (v 3.0.5) [39] and Chimera(v 1.19) [40]. High-resolution crystal structures of protein targets were retrieved from the pubchem and Zinc15 database. Chimera was used to remove water molecules and original ligands from protein structures. Subsequently, AutoDock was applied to hydrogenate the receptors (proteins) and perform molecular docking with small-molecule ligands. Binding activities were evaluated on the basis of binding energy. Finally, PyMOL was utilized to analyze and visualize the molecular docking results.

### 2.8. Molecular Dynamics Simulation

MD simulation is an approach used to evaluate the stability and dynamic interactions of proteins and/or between a protein and their ligands. Here, the GROMACS (v2025) MD simulation package was employed to conduct the MD simulation of proteins and their ligands. Protein topologies and the parameters were generated with the AMBER99SB force field, whereas ligand topologies and the parameters were obtained via the Chimera server. In this study, the structural properties evaluated were RMSD [41], radius of gyration (Rg) [42], solvent-accessible surface area (SASA) [43], RMSF [44], hydrogen bonding, secondary structure, and angular and distance measurements. Simulation trajectories were performed using CMD (v10.0.26100.2894), and plots were generated with DuIvyTools (v0.5.0) (https://duivytools.readthedocs.io/; accessed on 27 February 2026).

### 2.9. Single-Cell RNA Sequencing Analysis

Here, we selected the single-cell dataset GSE214695 (comprising 6 control samples, 6 CD colon samples, and 6 UC colon samples) [45], GSE254513 (comprising 12 control samples and 15 samples of gastritis) [46], GSE201153 (comprising 5 control samples and 5 samples of esophagitis) [47] for analysis. We performed rigorous data curation and downstream analysis of 46,700 single cells using Seurat R package (v5.3.0). Samples were then pooled together in the same object. Low-quality cells were then filtered out based on mitochondrial RNA percentage and number of genes per cell. Immunoglobulin (IG) genes were removed from all of the main cell types except B and plasma cells to reduce background noise. Dimensionality reduction was conducted by using the Uniform Manifold Approximation and Projection (UMAP) algorithm using the optimal number of PCs. Marker genes were utilized to define each subcluster within the main cell types. The FindAllMarkers function was used to identify marker genes using the default threshold parameters, excluding the min.pct and the thresh.use, both of which were set to 0.25.

### 2.10. Statistical Analysis

All statistical analyses were performed using R software (version 4.4.2). For transcriptomic datasets obtained from GEO, differential expression analysis was performed between disease and control groups using the limma package. The moderated *t*-test implemented in limma was used to estimate differential expression, and *p*-values were adjusted for multiple testing using the Benjamini–Hochberg false discovery rate (FDR) method. Differentially expressed genes were defined as those with adj.*p* < 0.05 and |log_2_FC| ≥ 1. For functional enrichment analyses, GO and KEGG terms with adj.*p* < 0.05 were considered statistically significant. For single-cell RNA-seq analysis, marker genes or differentially expressed genes among cell clusters were identified using the Wilcoxon rank-sum test, where applicable. Unless otherwise stated, adj.*p* < 0.05 was considered statistically significant.

## 3. Results

### 3.1. Toxicity Analysis of DMP, DEP, DOP, and ATBC on the Gastrointestinal System

In this study, the PubChem database was used to search for DMP, DEP, DOP, and ATBC to obtain their standard structures and SMILES sequences (Table S1 in Supplementary File S2). The systemic and organ-specific toxicity profiles of DMP, DEP, DOP, and ATBC were predicted using ADMETlab 3.0 and ProTox-II databases. Both platforms consistently indicated that these four compounds exhibit toxicity towards gastrointestinal organs, especially highlighting the potential for liver injury. Additional toxic endpoints predicted by consensus included ocular irritation, skin sensitization, hepatotoxicity, nephrotoxicity, and neurotoxicity. The ProTox-II database classified the overall toxicity levels of DMP, DEP, DOP, and ATBC as Grade 6, Grade 6, Grade 4, and Grade 4, respectively. Their predicted human oral median lethal doses (LD50s) were 6800 mg/kg, 6172 mg/kg, 1340 mg/kg, and 517 mg/kg, respectively.

### 3.2. Target Screening and Enrichment Analysis for DMP, DEP, DOP, and ATBC in CD

**Figure 1:**
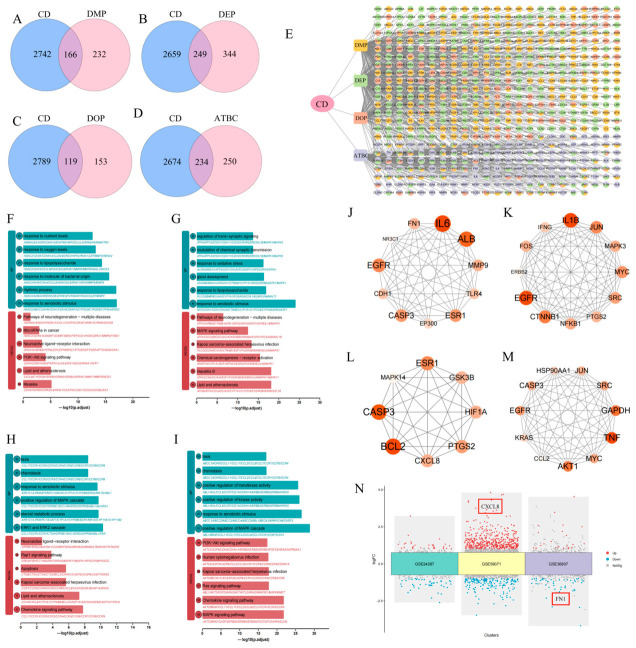
Screening of core targets shared by plasticizers and CD. (**A**–**D**) Overlapping targets between CD and each plasticizer, including DMP, DEP, DOP, and ATBC. (**E**) Cytoscape-based CD–target–plasticizer interaction network. (**F**–**I**) GO enrichment analysis of overlapping targets for each plasticizer–CD pair. (**J**–**M**) PPI-based hub target screening using DC, BC, and CC topological analyses. (**N**) Cross-analysis of hub targets with DEGs from GSE24287, GSE59071, and GSE36807 identified two significantly dysregulated core targets in CD. (adj.*p*  <  0.05, |log_2_FC|= > 1).

Potential targets for DMP (398), DEP (593), DOP (272), and ATBC (484) were identified by integrating data from the ChEMBL, PubChem, and SwissTargetPrediction databases, followed by the removal of duplicate entries. Concurrently, CD-associated targets (n = 2908) were obtained from the GeneCards, DrugBank, and OMIM databases. Venn diagram analysis identified 166, 249, 119, and 234 overlapping CD-related targets for DMP, DEP, DOP, and ATBC, respectively (Figure 1A–D). A disease–toxic compound–target network was then constructed (Figure 1E), and PPI networks of the intersecting targets were generated (Supplementary File S2, Figure S1). GO and KEGG enrichment analyses were performed separately for the overlapping targets of each plasticizer–CD pair (Figure 1F–I). DMP–CD targets were mainly enriched in response to xenobiotic stimulus and measles pathways; DEP–CD targets were associated with response to xenobiotic stimulus and lipid and atherosclerosis pathways; DOP–CD targets were related to the ERK1/2 cascade and chemokine signaling pathway; and ATBC–CD targets were enriched in positive regulation of the MAPK cascade and MAPK signaling pathway.

Subsequently, Cytoscape 3.8.2 was used to analyze the topological properties of the PPI networks, including degree centrality (DC), betweenness centrality (BC), and closeness centrality (CC). The top 15 genes ranked by each parameter were selected, and their intersections were visualized using Venn diagrams (Supplementary File S2, Figure S2). The intersecting hub genes were then used to construct network diagrams (Figure 1J–M). Finally, the four sets of hub targets were merged, deduplicated, and intersected with DEGs from the GSE24287, GSE59071, and GSE36807 datasets. This analysis identified two significantly differentially expressed core targets in CD, *CXCL8*, and *FN1* (Figure 1N).

### 3.3. Target Screening and Enrichment Analysis of DMP, DEP, DOP, and ATBC in UC

**Figure 2:**
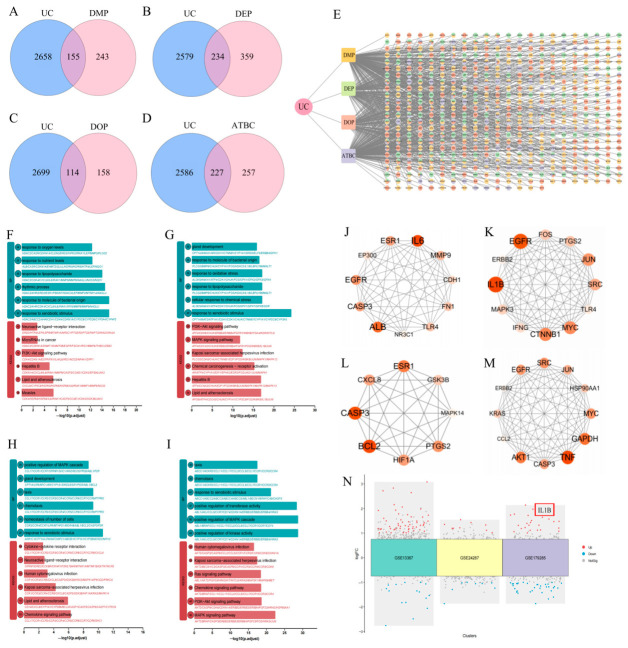
Screening of core targets shared by plasticizers and UC. (**A**–**D**) Overlapping targets between UC and each plasticizer, including DMP, DEP, DOP, and ATBC. (**E**) Cytoscape-based UC–target–plasticizer interaction network. (**F**–**I**) GO enrichment analysis of overlapping targets for each plasticizer–UC pair. (**J**–**M**) PPI-based hub target screening using DC, BC, and CC topological analyses. (**N**) Cross-analysis of hub targets with DEGs from GSE13367, GSE24287, and GSE179285 identified one significantly dysregulated core target in UC. (adj.*p* <  0.05, |log_2_FC|= > 1).

Venn diagram analysis identified 155, 234, 114, and 227 potential overlapping targets between DMP, DEP, DOP, and ATBC in UC, respectively (Figure 2A–D). The corresponding TTD network is presented in Figure 1E. Meanwhile, a PPI network of the intersecting targets was constructed (Figure S3 in Supplementary File S2).

Subsequently, the overlapping targets of DMP, DEP, DOP, and ATBC with UC were separately subjected to GO analysis and KEGG pathway analysis (Figure 2F–I). These analyses revealed that DMP-UC targets were mainly enriched in response to xenobiotic stimulus and Measles. DEP-UC targets were associated with response to xenobiotic stimulus and lipid and atherosclerosis. DOP-UC targets were mainly enriched in response to xenobiotic stimulus and Chemokine signaling pathway. ATBC-UC targets were mainly involved in positive regulation of kinase activity and MAPK signaling pathway. Cytoscape 3.8.2 was used to analyze the topological properties of the PPI networks, including DC, BC, and CC, the top 15 core genes were selected to generate a Venn diagram (Figure S4 in Supplementary File S2), PPI network analysis was conducted as described for CD (Figure 2J–M). Following merging and intersecting with DEGs from the GSE13367, GSE24287, and GSE179285 datasets, one core target showing significant differential expression in UC was identified: *IL1B* (Figure 2N).

### 3.4. Target Screening and Enrichment Analysis of DMP, DEP, DOP, and ATBC in Esophagitis

**Figure 3:**
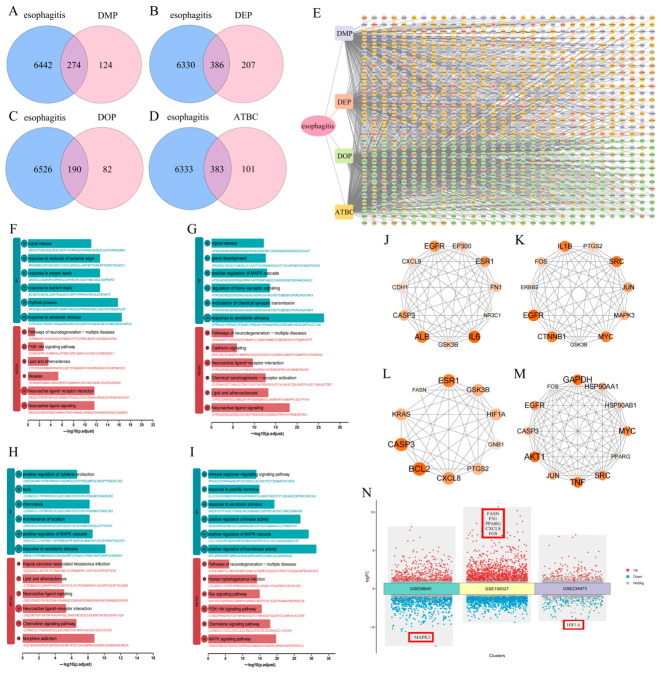
Screening of core targets shared by plasticizers and esophagitis. (**A**–**D**) Overlapping targets between esophagitis and each plasticizer, including DMP, DEP, DOP, and ATBC. (**E**) Cytoscape-based esophagitis–target–plasticizer interaction network. (**F**–**I**) GO enrichment analysis of overlapping targets for each plasticizer–esophagitis pair. (**J**–**M**) PPI-based hub target screening using DC, BC, and CC topological analyses. (**N**) Cross-analysis of hub targets with DEGs from GSE58640, GSE190027, and GSE234973 identified seven significantly dysregulated core targets in esophagitis. (adj.*p* <  0.05, |log_2_FC|= > 1).

Venn diagram analysis identified 274, 386, 190, and 383 potential overlapping targets for DMP, DEP, DOP, and ATBC in esophagitis, respectively (Figure 3A–D). A TTD network was shown in Figure 3E. Meanwhile, a PPI network of the intersecting targets was constructed (Figure S5 in Supplementary File S2). The overlapping targets of the four compounds with esophagitis were separately subjected to GO analysis and KEGG pathway analysis (Figure 3B–F). These analyses showed that the targets of DMP and DEP in esophagitis were mainly enriched in response to xenobiotic stimulus and neuroactive ligand–receptor interaction. Targets of DOP in esophagitis were mainly associated with response to xenobiotic stimulus and morphine addiction. Targets of ATBC in esophagitis were mainly enriched in positive regulation of transferase activity and MAPK signaling pathway. Subsequently, Cytoscape 3.8.2 was applied to analyze the topological properties of the PPI networks, including DC, BC, and CC, the top 15 core genes were selected to generate a Venn diagram (Figure S6 in Supplementary File S2) and the topological properties of the PPI networks were analyzed and visualized (Figure 3J–M). The four sets of overlapping targets were then merged and deduplicated, followed by intersection analysis with DEGs from the GSE58640, GSE190027, and GSE234973 datasets. Seven core targets showing significant differential expression in esophagitis were identified: *MAPK3*, *FASN*, *FN1*, *PPARG*, *CXCL8*, *FOS*, and *HIF1A*. The screening results were visualized using multiple grouped bar–volcano plots (Figure 3N).

### 3.5. Target Screening and Enrichment Analysis of DMP, DEP, DOP, and ATBC in Gastritis

**Figure 4:**
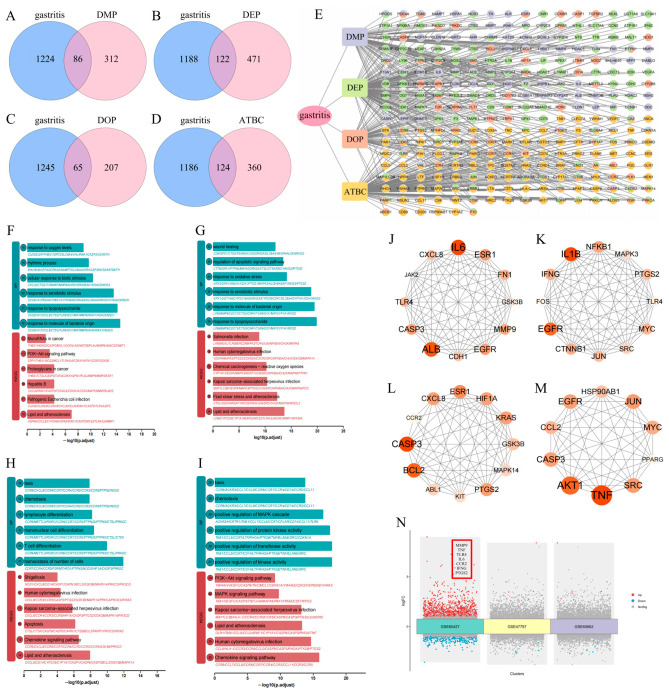
Screening of core targets shared by plasticizers and gastritis. (**A**–**D**) Overlapping targets between gastritis and each plasticizer, including DMP, DEP, DOP, and ATBC. (**E**) Cytoscape-based gastritis–target–plasticizer interaction network. (**F**–**I**) GO enrichment analysis of overlapping targets for each plasticizer–gastritis pair. (**J**–**M**) PPI-based hub target screening using DC, BC, and CC topological analyses. (**N**) Cross-analysis of hub targets with DEGs from GSE60427, GSE47797, and GSE60662 identified seven significantly dysregulated core targets in gastritis. (adj.*p*  <  0.05, |log_2_FC|= > 1).

A total of 274, 386, 190, and 383 potential overlapping targets were identified for DMP, DEP, DOP, and ATBC in gastritis, respectively (Figure 4A–D). A TTD network was constructed and presented in Figure 4E. Meanwhile, a PPI network of the intersecting targets was constructed (Figure S7 in Supplementary File S2).

The overlapping targets of DMP, DEP, DOP, and ATBC with gastritis were separately subjected to GO analysis and KEGG pathway analysis (Figure 4F–I). Enrichment analysis revealed distinct pathways: Targets of DMP in gastritis were mainly enriched in response to molecules of bacterial origin and lipid/atherosclerosis pathways. Targets of DEP were mainly associated with response to lipopolysaccharide and lipid/atherosclerosis. DOP targets were mainly involved in cell homeostasis and lipid/atherosclerosis. Targets of ATBC were mainly enriched in positive regulation of kinase activity and the chemokine signaling pathway. Then, Cytoscape 3.8.2 was used to analyze the topological properties of the PPI networks, including DC, BC, and CC, the top 15 core genes were selected to generate a Venn diagram (Figure S8 in Supplementary File S2), and the intersecting genes were used to construct a network diagram (Figure 4J–M). Integration with DEGs from the GSE60427, GSE47797, and GSE60662 datasets identified seven core targets with significant differential expression in gastritis: *MMP9*, *TNF*, *TLR4*, *IL6*, *CCR2*, *IFNG*, and *PTGS2* (Figure 4N).

### 3.6. Common Target Analysis of DMP in IGDs

**Figure 5:**
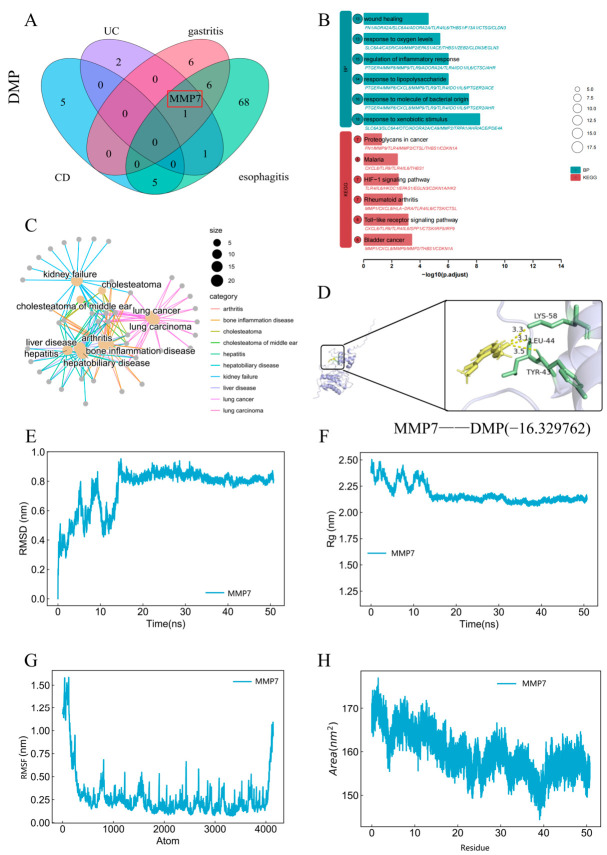
Identification of common DMP-associated target across IGDs. (**A**) Venn diagram showing the shared differential targets of DMP with CD, UC, esophagitis, and gastritis, respectively. (**B**) GO and KEGG enrichment analyses of the shared differential targets. (**C**) DO enrichment analysis of the shared differential targets. (**D**) Molecular docking diagram of MMP7 with DMP. (**E**–**H**) Molecular dynamics simulation diagrams of MMP7 with DMP.

We determined the common differentially expressed target genes of DMP in CD, UC, esophagitis, and gastritis by taking the intersection. *MMP7* was identified as the sole target consistently dysregulated in three or more of these conditions (Figure 5A). Functional enrichment analyses (GO, KEGG, and Disease Ontology) indicated that the shared targets were significantly enriched in biological processes related to response to xenobiotic stimulus and pathways associated with bladder cancer (Figure 5B). Moreover, these targets exhibited strong associations with various inflammatory and organ-specific diseases, including rheumatoid arthritis, cholesteatoma, cholesteatoma of middle ear, viral hepatitis, hepatobiliary diseases, acute kidney failure, chronic liver diseases, and non-small cell lung cancer (Figure 5C). To further explore the binding affinity between MMP7 and DMP, molecular docking simulations (Figure 5D) and 100-ns-atom molecular dynamics simulations (Figure 5E–H) were performed, confirming stable binding and favorable binding affinity.

### 3.7. Common Target Analysis of DEP in IGDs

**Figure 6:**
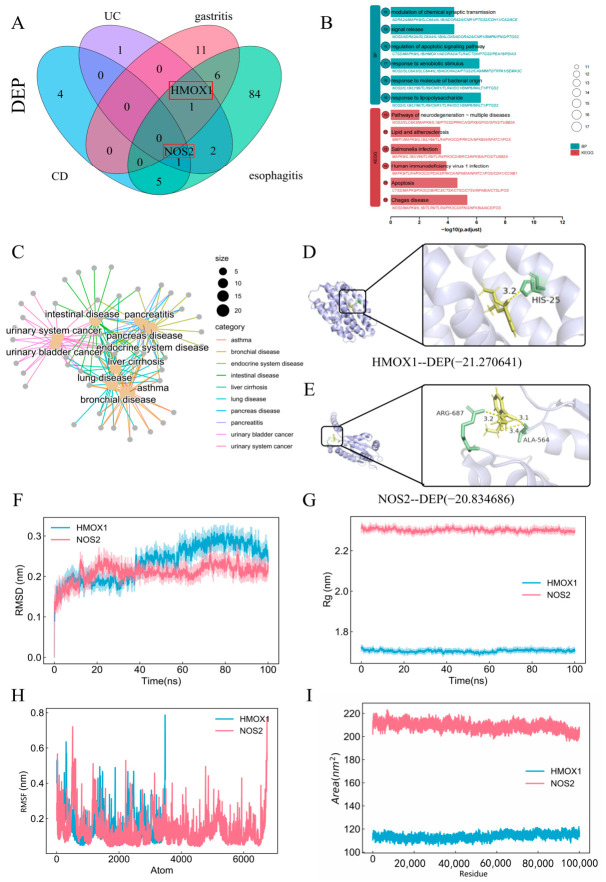
Identification of common DEP-associated targets across IGDs. (**A**) Venn diagram showing the shared differential targets of DEP with CD, UC, esophagitis and gastritis, respectively. (**B**) GO and KEGG enrichment analyses of the shared differential targets. (**C**) DO enrichment analysis of the shared differential targets. (**D**) Molecular docking diagram of HMOX1 with DEP. (**E**) Molecular docking diagram of NOS2 with DEP. (**F**–**I**) Molecular dynamics simulation diagrams of HMOX1 and NOS2 with DEP.

Shared differential targets of DEP in at least three IGDs were identified and are visualized using a Venn diagram (Figure 6A). *HMOX1* and *NOS2* were found to be the only two targets consistently dysregulated in three or more of the four IGDs. Subsequently, GO functional enrichment, KEGG pathway enrichment (Figure 6B), and Disease Ontology (DO) analysis (Figure 6C) were conducted on these common differential targets. These targets were mainly enriched in response to lipopolysaccharide and Chagas disease, and were associated with a spectrum of diseases including asthma, bronchial diseases, endocrine system diseases, intestinal diseases, liver cirrhosis, lung diseases, pancreas diseases, pancreatitis, urinary bladder cancer, and urinary system cancers. To characterize the binding affinity between DEP and its top candidate targets, molecular docking (Figure 6D,E) and molecular dynamics simulations (Figure 6F–I) were further performed. Collectively, these results indicate that DEP forms a thermodynamically stable complex with HMOX1, exhibiting favorable binding affinity and sustained intermolecular interactions throughout the simulation trajectory.

### 3.8. Common Target Analysis of DOP Targets in IGDs

**Figure 7:**
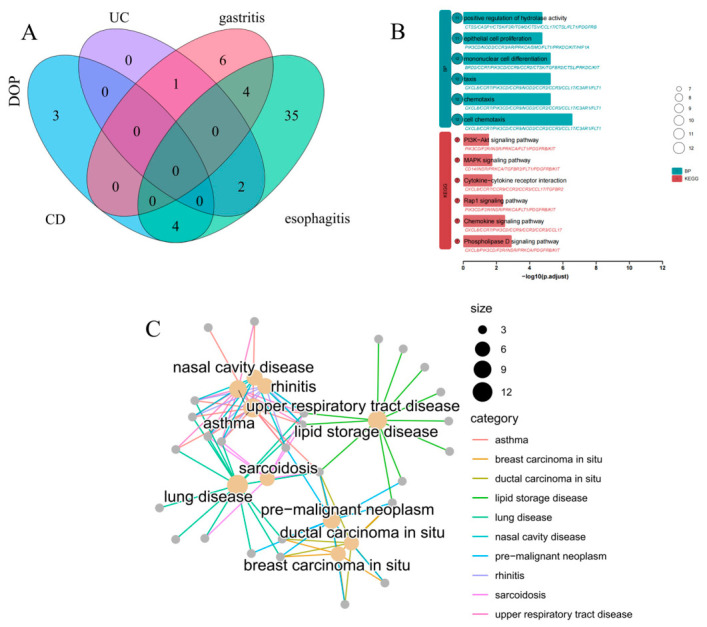
Identification of common DOP-associated target across IGDs. (**A**) Venn diagram showing the shared differential targets of DOP with CD, UC, esophagitis and gastritis, respectively. (**B**) GO and KEGG enrichment analyses of the shared differential targets. (**C**) DO enrichment analysis of the shared differential targets.

Notably, DOP does not share any common target molecules in at least three IGDs, and this has been visually demonstrated using a Venn diagram (Figure 7A). Consequently, GO functional enrichment, KEGG pathway enrichment (Figure 7B), and DO analyses (Figure 7C) were performed exclusively on this set of shared targets. The results indicated significant association with biological processes such as cell chemotaxis and phospholipase D signaling pathway. Moreover, DO analysis revealed statistically enriched associations with a spectrum of non-GD conditions, including asthma, breast carcinoma in situ, ductal carcinoma in situ, lipid storage disease, lung disease, nasal cavity disease, pre-malignant neoplasm, rhinitis, sarcoidosis, and upper respiratory tract disease.

### 3.9. Common Target Analysis of ATBC in IGDs

**Figure 8:**
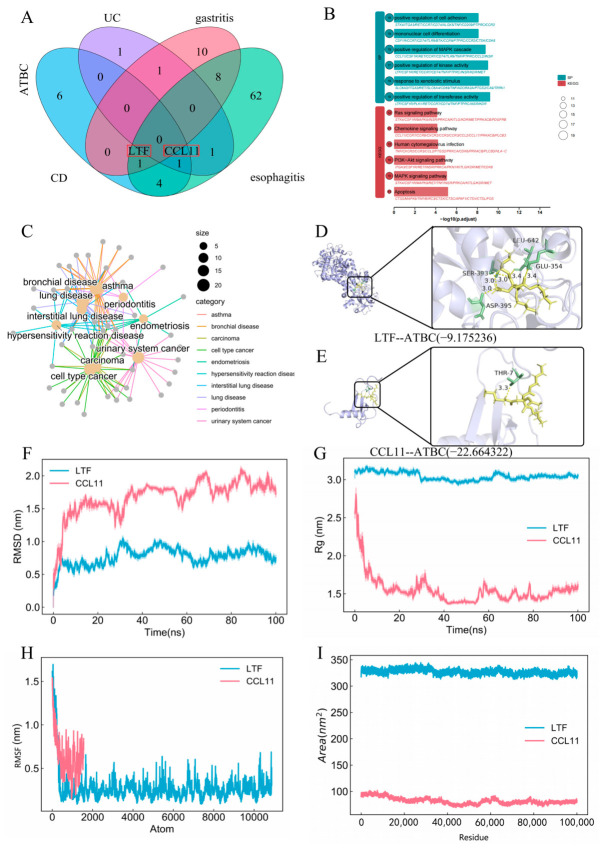
Identification of common ATBC-associated target across IGDs. (**A**) Venn diagram showing the shared differential targets of ATBC with CD, UC, esophagitis and gastritis, respectively. (**B**) GO and KEGG enrichment analyses of the shared differential targets. (**C**) DO enrichment analysis of the shared differential targets. (**D**) Molecular docking diagram of LTF with ATBC. (**E**) Molecular docking diagram of CCL11 with ATBC. (**F**–**I**) Molecular dynamics simulation diagrams of LTF and CCL11 with ATBC.

Shared differential targets of DEP in at least three IGDs were identified and are visualized using a Venn diagram (Figure 8A). *LTF* and *CCL11* were identified as the two targets commonly dysregulated across three or more diseases. Functional characterization of these common targets was subsequently performed via GO functional enrichment, KEGG pathway enrichment (Figure 8B), and DO enrichment analysis (Figure 8C). GO analysis revealed significant enrichment in biological processes, including positive regulation of transferase activity and apoptosis. DO analysis revealed that the common targets associated with a spectrum of pathologies, including asthma, bronchial disease, carcinoma, cell type cancer, endometriosis, hypersensitivity reaction disease, interstitial lung disease, lung disease, periodontitis, and urinary system cancer. To investigate the binding affinity between ATBC and the top candidate targets, molecular docking (Figure 8D,E) and molecular dynamics simulations (Figure 8F–I) were further performed. These computational analyses demonstrate that CCL11 exhibits robust and stable binding affinity toward ATBC, suggesting its potential role as a key mechanistic mediator in ATBC’s therapeutic effects.

### 3.10. Cell-Type Specific Expression Profiling of Key Disease-Associated and Toxicant-Associated Targets in Diseased Tissues

**Figure 9:**
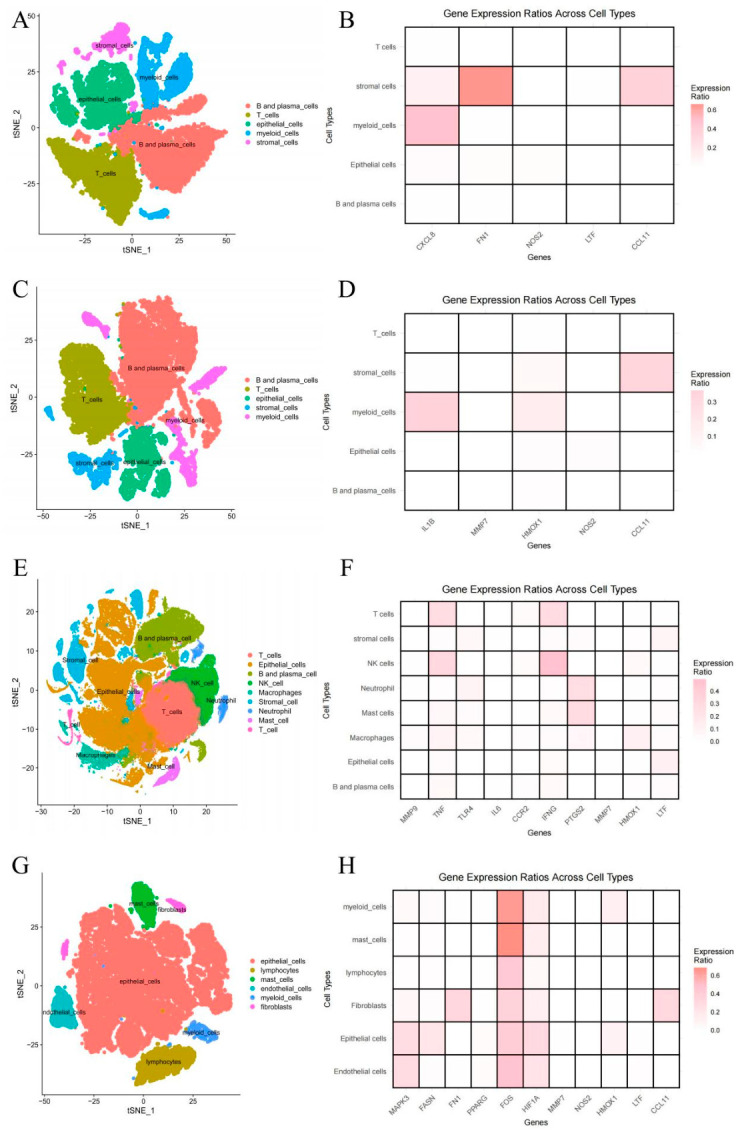
Single-cell analysis of different IGD organizations. Cell type annotations (**A**,**C**,**E**,**G**) and heatmaps (**B**,**D**,**F**,**H**) illustrating the distribution of disease-specific targets and toxicant-specific targets across different cell types.

Single-cell RNA sequencing (scRNA-seq) analyses were performed across three independent inflammatory gastrointestinal disease cohorts to characterize the cellular context-specific expression of key disease-associated targets and key toxicant-associated targets identified in prior network pharmacology and toxicity screening steps. First, scRNA-seq data from the GSE214695 dataset, comprising six healthy control samples, six CD patient samples, and six UC patient samples, were processed and clustered. Cell-type annotation revealed conserved major lineages, such as epithelial cells, T cells, macrophages, and stromal cells, across samples. Corresponding cell type distribution maps for CD and control groups are shown in Figure 9A. Heatmap visualization of target gene expression across annotated cell types demonstrated that *FN1* exhibited extremely high expression in stromal cells in CD tissues (Figure 9B), a finding consistent with its established role in extracellular matrix remodeling and fibro-inflammatory activation. Parallel analysis of UC versus control samples (Figure 9C) revealed broadly attenuated expression of all signature targets across immune and structural cell populations, as summarized in Figure 9D.

Second, scRNA-seq data from the gastritis cohort (GSE254513, 12 controls, 15 gastritis samples) were analyzed. Cell-type composition maps for gastritis and control samples are presented in Figure 9E. Heatmap-based expression profiling identified *IFNG* as significantly enriched in NK cells (Figure 9F), suggesting a potential NK-mediated immunomodulatory axis in gastric mucosal inflammation.

Third, scRNA-seq data from the esophagitis cohort (GSE201153, five controls, five esophagitis samples) were integrated and annotated. Cell type distribution maps for esophagitis and control samples are displayed in Figure 9G. As shown in Figure 9H, *FOS*, a core immediate early transcription factor, exhibited broad, high-level expression across various epithelial and immune cell types (Figure 9H), indicating widespread transcriptional activation in response to esophageal injury or inflammation.

Collectively, these cross-disease, cell-type specific analyses delineate distinct functional contexts for the prioritized targets, supporting their biological relevance and informing hypothesis-driven mechanistic validation.

## 4. Discussion

Plasticizers constitute a class of chemical additives extensively employed in polymeric materials to enhance processability, flexibility, and mechanical durability. By reducing intermolecular forces within polymer matrices, they improve material pliability and impact resilience, thereby expanding the utility of plastic products across diverse sectors, including medical devices, food packaging, and personal care products [48,49]. The widespread application of these additives has led to pervasive environmental and subsequent human exposure via inhalation and ingestion [49]. In this study, we established an integrative network toxicology framework to systematically investigate the potential molecular links between four representative plasticizers—DMP, DEP, DOP/DEHP [1,50], and ATBC [51,52]—and four IGDs, including CD, UC, esophagitis, and gastritis. By integrating target prediction, disease-associated gene collection, PPI network analysis, transcriptomic validation, molecular docking, molecular dynamics simulation, and single-cell RNA-seq analysis, we identified both disease-specific and plasticizer-associated candidate targets. These findings suggest that plasticizer-related compounds may influence gastrointestinal inflammation through multiple biological processes, including xenobiotic response, chemokine signaling, MAPK-related stress responses, extracellular matrix remodeling, epithelial injury, and immune-cell activation. [53,54,55,56,57].

Our disease-specific analysis identified *CXCL8* and *FN1* as candidate targets associated with CD, *IL1B* with UC, *MAPK3*, *FASN*, *FN1*, *PPARG*, *CXCL8*, *FOS*, and *HIF1A* with esophagitis, and *MMP9*, *TNF*, *TLR4*, *IL6*, *CCR2*, *IFNG*, and *PTGS2* with gastritis. These targets are biologically consistent with the known pathological features of inflammatory gastrointestinal diseases. *CXCL8* is a central pro-inflammatory chemokine involved in neutrophil recruitment and mucosal immune activation and has been reported in CD, UC, gastritis, and esophagitis [58,59,60,61]. This is consistent with our finding that *CXCL8* may represent a shared signature target across several IGDs. Notably, a previous clinical study involving pediatric patients with eosinophilic esophagitis (EoE) demonstrated that *CXCL8* is one of the few significantly upregulated biomarkers in active EoE compared with healthy controls, and its elevation is strictly dependent on ex vivo immune stimulation [58]. *FN1*, an extracellular matrix glycoprotein, may reflect stromal activation, tissue remodeling, and fibro-inflammatory responses, particularly in CD [62,63,64]. *IL1B* is a key inflammasome-related cytokine and an important mediator of intestinal inflammation in IBD [65]. In esophagitis, *MAPK3* and *FOS* may indicate stress-response and inflammatory transcriptional activation, whereas *HIF1A* and *PPARG* may participate in epithelial injury, metabolic adaptation, and inflammatory regulation [66,67]. In gastritis, *TNF*, *TLR4*, *IL6*, *IFNG*, *PTGS2*, *MMP9*, and *CCR2* represent inflammatory cytokine signaling, innate immune activation, monocyte recruitment, and mucosal tissue remodeling [68,69,70,71,72]. Together, these findings indicate that plasticizer-associated molecular perturbations may converge on inflammatory and remodeling pathways that are central to gastrointestinal disease progression.

These findings are partly consistent with previous experimental studies on plasticizer-induced gastrointestinal toxicity. Increasing evidence suggests that chronic or repeated exposure to plasticizers may compromise intestinal epithelial barrier function, alter gut microbiota composition, induce oxidative stress, and trigger inflammatory signaling pathways, thereby contributing to intestinal toxicity and gastrointestinal dysfunction [73,74,75]. In addition, although ATBC is widely used as a phthalate-free alternative plasticizer, recent evidence suggests that chronic ATBC exposure may also induce intestinal toxicity and inflammatory responses. This supports our finding that ATBC-associated targets, such as *LTF* and *CCL11*, may be involved in mucosal immune regulation and inflammatory processes. However, the current literature remains limited, particularly regarding long-term human exposure, dose–response relationships, and direct evidence linking ATBC exposure to inflammatory gastrointestinal diseases. Therefore, our computational results are consistent with some reported toxicological effects, but they should be interpreted as mechanistic hypotheses rather than definitive causal evidence.

Cross-disease toxicant-specific analysis further identified *MMP7* as a DMP-associated target, *HMOX1* and *NOS2* as DEP-associated targets, and *LTF* and *CCL11* as ATBC-associated targets. These targets suggest that different plasticizers may affect IGDs through partially distinct mechanisms. *MMP7* is involved in extracellular matrix degradation, epithelial repair, and inflammatory tissue remodeling, suggesting that DMP may be linked to mucosal remodeling processes [76]. *HMOX1* and *NOS2* are closely related to oxidative stress, nitric oxide metabolism, and inflammatory responses, supporting a potential role of DEP in redox imbalance and immune activation [77]. *LTF* and *CCL11* are associated with antimicrobial defense, epithelial immunity, eosinophil recruitment, and allergic-type inflammation, suggesting that ATBC may be connected with mucosal immune modulation [78]. Notably, DOP/DEHP did not show a common target shared across three or more diseases, implying that its potential effects may be more disease-specific or mediated through heterogeneous molecular pathways rather than a single convergent target.

Molecular docking and molecular dynamics simulations provided additional structural-level support for the predicted toxicant–target relationships. The stable binding patterns observed between selected plasticizers and their candidate targets suggest that these compounds may have the potential to interact with key inflammatory or remodeling-related proteins. However, these results should be interpreted as computational evidence of binding feasibility rather than direct proof of toxicological causality. Docking and dynamics simulations cannot determine whether plasticizers regulate gene expression, protein activity, or downstream signaling in biological systems. Therefore, the predicted interactions require further validation using cellular models, animal experiments, and exposure-response assays.

The single-cell RNA-seq analysis further strengthened the biological relevance of the prioritized targets by revealing their cell-type-specific expression patterns in diseased tissues. In CD tissues, *FN1* showed high expression in stromal cells, supporting its role in extracellular matrix remodeling and fibro-inflammatory activation. In gastritis, *IFNG* was enriched in NK cells, suggesting that NK-cell-mediated immune responses may contribute to gastric mucosal inflammation. In esophagitis, *FOS* was broadly expressed across epithelial and immune cell populations, indicating widespread transcriptional activation under inflammatory or injury-related conditions. These observations provide cellular context for the network toxicology findings and suggest that plasticizer-associated targets may act within specific mucosal microenvironmental compartments, including stromal, epithelial, and immune-cell niches.

This study has several strengths. First, we established an integrative network toxicology framework combining public target prediction, disease-related target collection, PPI network analysis, topological screening, functional enrichment analysis, transcriptomic validation, molecular docking, molecular dynamics simulation, and single-cell RNA-seq analysis. This multi-step strategy allowed us to systematically identify disease-specific and plasticizer-associated candidate targets across multiple inflammatory gastrointestinal disorders. Second, by integrating multiple GEO datasets and single-cell transcriptomic data, this study provided both transcriptomic and cell-type-specific evidence to support the biological relevance of the predicted targets. Third, we systematically evaluated the potential toxicological mechanisms as well as the similarities and differences of plasticizer exposure in inflammatory gastrointestinal disorders.

This study also has several limitations. First, the findings are mainly based on public databases, computational target prediction, and transcriptomic integration; toxicological targets predicted via ChEMBL, PubChem and SwissTargetPrediction and disease-related targets identified using GeneCards, DrugBank and OMIM are all in silico predictions, which may lead to false positive outcomes. By contrast, datasets from the GEO database are derived from experimental evidence. Therefore, they cannot establish direct causal relationships between plasticizer exposure and disease progression. Second, molecular docking and molecular dynamics simulations only evaluate predicted binding affinity and structural stability, but do not confirm actual protein activity changes or downstream pathway activation. Third, the GEO and single-cell datasets used in this study do not contain direct measurements of plasticizer exposure, exposure duration, tissue concentration, or metabolite levels. Fourth, the combined effects of mixed plasticizer exposure were not evaluated, although real-world exposure usually involves multiple compounds simultaneously. Finally, due to limitations in time, funding, and experimental resources, no in vitro or in vivo validation experiments (e.g., ELISA, Western blotting, cell-based assays, or animal studies) were performed in the current study. Therefore, the proposed mechanisms and the potential roles of *CXCL8*, *FN1*, *HMOX1*, *NOS2*, *LTF*, and *CCL11* should be considered preliminary and require further experimental validation in future studies.

Future studies should further validate these candidate targets and pathways using intestinal and gastric epithelial cell models, immune-cell co-culture systems, and animal models of gastrointestinal inflammation under chronic plasticizer exposure. In addition, population-based studies with direct measurements of plasticizer metabolites, exposure duration, and dose–response relationships are needed to clarify the relevance of these findings in humans. The combined effects of mixed plasticizer exposure should also be investigated, as real-world exposure usually involves multiple compounds simultaneously.

## 5. Conclusions

In conclusion, this study provides a systematic computational framework for exploring the potential molecular associations between plasticizer exposure and IGDs. By identifying disease-specific and plasticizer-associated molecular targets and integrating transcriptomic, structural, and single-cell evidence, our findings offer mechanistic clues for understanding how plasticizer-related compounds may be involved in gastrointestinal inflammation. These results may help prioritize candidate biomarkers and molecular targets for future toxicological validation, environmental risk assessment, and prevention strategies for IGDs.

## Data Availability

mRNA expression profiles were obtained from the GEO database (https://www.ncbi.nlm.nih.gov/geo/) (accessed on 23 March 2026).

## Supplementary Materials

The following supporting information can be downloaded at https://www.mdpi.com/article/10.3390/genes17060667/s1. Supplementary File S1: Toxicity report and DEG list. Supplementary File S2: Table S1: The structures of the compounds. Figures S1, S3, S5 and S7: (a–d) PPI network of overlapping targets. Figures S2, S4, S6 and S8: (a–d) Venn diagram showing the intersection of the top 15 targets selected using three local topology methods (DC, BC, CC).
