# Supplementary material for: Integrative Network Toxicology Reveals Potential Molecular Targets Linking Plasticizer Exposure to Inflammatory Gastrointestinal Disorders
Source: Genes (Basel). 2026 Jun 7;17(6):667. doi: 10.3390/genes17060667 (PMC13299468; doi:10.3390/genes17060667)
Supplement: Supplementary file 1 [file genes-17-00667-s001.zip › Supplementary File S1/Diocytl phthalate/ADMETlab3_result_DOP.pdf]

1. Physicochemical Property

Property	Value	Comment
Molecular Weight	390.28	Contain hydrogen atoms. Optimal:100~600
Volume	437.082	Van der Waals volume
Density	0.893	Density = MW / Volume
nHA	4.0	Number of hydrogen bond acceptors. Optimal:0~12
nHD	0.0	Number of hydrogen bond donors. Optimal:0~7
nRot	18.0	Number of rotatable bonds. Optimal:0~11
nRing	1.0	Number of rings. Optimal:0~6
MaxRing	6.0	Number of atoms in the biggest ring. Optimal:0~18
nHet	4.0	Number of heteroatoms. Optimal:1~15
fChar	0.0	Formal charge. Optimal:-4 ~4
nRig	8.0	Number of rigid bonds. Optimal:0~30
Flexibility	2.25	Flexibility = nRot /nRig
Stereo Centers	0.0	Stereo Centers. Optimal: ≤ 2
TPSA	52.6	Topological Polar Surface Area. Optimal:0~140
logS	-6.751	The logarithm of aqueous solubility value.
logP	7.218	The logarithm of the n-octanol/water distribution coefficients at pH=7.4.
logD	4.133	The logarithm of the n-octanol/water distribution coefficient.
pka (Acid)	8.688	Acid-base dissociation constant (pKa) value represents the strength of a drug molecule's acidity or basicity.
pka (Base)	3.487	Acid-base dissociation constant (pKa) value represents the strength of a drug molecule's acidity or basicity.
Melting point	-4.38	The predicted melting point of a compound is expressed in degrees Celsius (°C).<br>Melting points below 25°C are classified as liquids, while melting points above 25°C are classified as solids.
Boiling point	385.803	The predicted melting point of a compound is expressed in degrees Celsius (°C).<br>A normal boiling point below 25°C is categorized as a gas.

2. Medicinal Chemistry

Property	Value	Decision	Comment

ADMETlab 3.0

QED	0.232	🔴	■ A measure of drug-likeness based on the concept of desirability;<br>■ Attractive: > 0.67;<br>■ unattractive: 0.49~0.67;<br>■ too complex: < 0.34
GASA	0.0	🟢	■ ES: Easy to synthesize; HS: Hard to synthesize;<br>■ The output value represents the probability of being difficult to synthesize, ranging from 0 to 1.
Synth	1.0	🟢	■ Synthetic accessibility score is designed to estimate ease of synthesis of drug-like molecules.<br>■ SAscore ≥ 6, difficult to synthesize; SAscore <6, easy to synthesize
Fsp3	0.667	🟢	■ The number of sp3 hybridized carbons / total carbon count, correlating with melting point and solubility.<br>■ Fsp^3 ≥0.42 is considered a suitable value.
MCE-18	7.0	🔴	■ MCE-18 stands for medicinal chemistry evolution.<br>■ MCE-18≥45 is considered a suitable value.
NPscore	-0.078	-	■ Natural product-likeness score.<br>■ This score is typically in the range from -5 to 5.<br>■ The higher the score is, the higher the probability is that the molecule is a NP.
Lipinski Rule	0.0	🟢	■ MW ≤ 500; logP ≤ 5; Hacc ≤ 10; Hdon ≤ 5<br>■ If two properties are out of range, a poor absorption or permeability is possible, one is acceptable.
Pfizer Rule	1.0	🔴	■ logP > 3; TPSA < 75<br>■ Compounds with a high log P (>3) and low TPSA (<75) are likely to be toxic.
GSK Rule	1.0	🔴	■ MW ≤ 400; logP ≤ 4<br>■ Compounds satisfying the GSK rule may have a more favorable ADMET profile
Golden Triangle	0.0	🟢	■ 200 ≤ MW ≤ 500; -2 ≤ logD ≤ 5<br>■ Compounds satisfying the Golden Triangle rule may have a more favorable ADMET profile.
PAINS	0 alerts	-	frequent hitters, Alpha-screen artifacts and reactive compound 480 substructures (J Med Chem 201053:2719-40)
ALARM NMR	0 alerts	-	Thiol reactive compounds.
BMS	0 alerts	-	undesirable, reactive compounds 176 substructures (J Chem Inf Model 200646:1060-8)
Chelator Rule	0 alerts	-	Chelating compounds.
Colloidal aggregators	0.568	-	■ Category 0: non-colloidal aggregators;<br>■ Category 1: colloidal aggregators.<br>■ The output value is the probability of being colloidal aggregators, within the range of 0 to 1.

FLuc inhibitors	0.293	●	■ Category 0: non-fLuc inhibitors;<br>■ Category 1: fLuc inhibitors.<br>■ The output value is the probability of being fLuc inhibitors, within the range of 0 to 1.
Blue fluorescence	0.022	●	■ Category 0: non-blue fluorescence;<br>■ Category 1: blue fluorescence.<br>■ The output value is the probability of being blue fluorescence, within the range of 0 to 1.
Green fluorescence	0.07	●	■ Category 0: non-green fluorescence;<br>■ Category 1: green fluorescence.<br>■ The output value is the probability of being green fluorescence, within the range of 0 to 1.
Reactive compounds	0.909	●	■ Category 0: non-reactive compound;<br>■ Category 1: reactive compound.<br>■ The output value is the probability of being reactive compound, within the range of 0 to 1.
Promiscuous compounds	0.138	●	■ Category 0: non-promiscuous compound;<br>■ Category 1: promiscuous compound.<br>■ The output value is the probability of being promiscuous compound, within the range of 0 to 1.

3. Absorption

Property	Value	Decision	Comment
Caco-2 Permeability	-5.084	●	Optimal: higher than -5.15 Log unit
MDCK Permeability	-4.829	●	■ low permeability: < 2 × 10-6 cm/s<br>■ medium permeability: 2-20 × 10^{-6} cm/s<br>■ high passive permeability: > 20 × 10^{-6} cm/s
PAMPA	0.001	●	■ The experimental data for Peff was logarithmically transformed (logPeff).<br>■ Molecules with log Peff values below 2.0 were classified as low-permeability (Category 0), while those with log Peff values exceeding 2.5 were classified as high-permeability (Category 1).
Pgp-inhibitor	0.01	●	■ Category 1: Inhibitor;<br>■ Category 0: Non-inhibitor;<br>■ The output value is the probability of being Pgp-inhibitor
Pgp-substrate	0.009	●	■ Category 1: substrate;<br>■ Category 0: Non-substrate;<br>■ The output value is the probability of being Pgp-substrate
HIA	0.086	●	■ Human Intestinal Absorption<br>■ Category 1: HIA+(HIA < 30%);<br>■ Category 0: HIA-(HIA >= 30%);<br>■ The output value is the probability of being HIA+

ADMETlab 3.0

$F_{20\%}$	0.832	●	■ 20% Bioavailability<br>■ Category 1: F 20% + (bioavailability < 20%);<br>■ Category 0: F 20% - (bioavailability ≥ 20%);<br>■ The output value is the probability of being F 20% +
$F_{30\%}$	0.951	●	■ 30% Bioavailability<br>■ Category 1: F 30% + (bioavailability < 30%);<br>■ Category 0: F 30% - (bioavailability ≥ 30%);<br>■ The output value is the probability of being F 30% +
$F_{50\%}$	0.483	●	■ 50% Bioavailability<br>■ Category 1: F 50% + (bioavailability < 50%);<br>■ Category 0: F 50% - (bioavailability ≥ 50%);<br>■ The output value is the probability of being F 50% +

4. Distribution

Property	Value	Decision	Comment
PPB	99.269	●	■ Plasma Protein Binding<br>Optimal: < 90%.<br>■ Drugs with high protein-bound may have a low therapeutic index.
VDss	1.254	●	■ Volume Distribution<br>■ Optimal: 0.04-20L/kg
BBB	0.059	●	■ Blood-Brain Barrier Penetration<br>■ Category 1: BBB+; Category 0: BBB-;<br>■ The output value is the probability of being BBB+
Fu	1.151	●	■ The fraction unbound in plasms<br>■ Low: <5%; Middle: 5~20%; High: > 20%
OATP1B1 inhibitor	0.458	●	■ Category 0: Non-inhibitor; Category 1: inhibitor.<br>■ The output value is the probability of being inhibitor, within the range of 0 to 1.
OATP1B3 inhibitor	1.0	●	■ Category 0: Non-inhibitor; Category 1: inhibitor.<br>■ The output value is the probability of being inhibitor, within the range of 0 to 1.
BCRP inhibitor	0.213	●	■ Category 0: Non-inhibitor; Category 1: inhibitor.<br>■ The output value is the probability of being inhibitor, within the range of 0 to 1.
MRP1 inhibitor	0.996	●	■ Category 0: Non-inhibitor; Category 1: inhibitor.<br>■ The output value is the probability of being inhibitor, within the range of 0 to 1.

5. Metabolism

Property	Value	Decision	Comment
CYP1A2 inhibitor	1.0	●	■ Category 1: Inhibitor; Category 0: Non-inhibitor;<br>■ The output value is the probability of being inhibitor.

ADMETlab 3.0

CYP1A2 substrate	0.0	●	■ Category 1: Substrate; Category 0: Non-substrate; ■ The output value is the probability of being substrate.
CYP2C19 inhibitor	1.0	●	■ Category 1: Inhibitor; Category 0: Non-inhibitor; ■ The output value is the probability of being inhibitor.
CYP2C19 substrate	0.0	●	■ Category 1: Substrate; Category 0: Non-substrate; ■ The output value is the probability of being substrate.
CYP2C9 inhibitor	0.24	●	■ Category 1: Inhibitor; Category 0: Non-inhibitor; ■ The output value is the probability of being inhibitor.
CYP2C9 substrate	0.021	●	■ Category 1: Substrate; Category 0: Non-substrate; ■ The output value is the probability of being substrate.
CYP2D6 inhibitor	0.985	●	■ Category 1: Inhibitor; Category 0: Non-inhibitor; ■ The output value is the probability of being inhibitor.
CYP2D6 substrate	0.116	●	■ Category 1: Substrate; Category 0: Non-substrate; ■ The output value is the probability of being substrate.
CYP3A4 inhibitor	0.384	●	■ Category 1: Inhibitor; Category 0: Non-inhibitor; ■ The output value is the probability of being inhibitor.
CYP3A4 substrate	0.376	●	■ Category 1: Substrate; Category 0: Non-substrate; ■ The output value is the probability of being substrate.
CYP2B6 inhibitor	1.0	●	■ Category 1: Inhibitor; Category 0: Non-inhibitor; ■ The output value is the probability of being inhibitor.
CYP2B6 substrate	0.0	●	■ Category 1: Substrate; Category 0: Non-substrate; ■ The output value is the probability of being substrate.
CYP2C8 inhibitor	1.0	●	■ Category 1: Inhibitor; Category 0: Non-inhibitor; ■ The output value is the probability of being inhibitor.
HLM Stability	0.275	●	■ human liver microsomal (HLM) stability ■ Category 0: stable+ (HLM > 30 min); Category 1: unstable- (HLM ≤ 30 min). The output value is the probability of human liver microsomal instability, where a value closer to 1 indicates a higher likelihood of instability. The range is between 0 and 1.

6. Excretion

Property	Value	Decision	Comment

ADMETlab 3.0

CL_{plasma}	5.109	●	■ The unit of predicted CLplasma penetration is ml/min/kg. >15 ml/min/kg: high clearance; 5-15 ml/min/kg: moderate clearance; < 5 ml/min/kg: low clearance.
$T_{1/2}$	0.405	●	■ The unit of predicted T1/2 is hours.<br>■ ultra-short half-life drugs: 1/2 < 1 hour; short half-life drugs: T1/2 between 1-4 hours; intermediate short half-life drugs: T1/2 between 4-8 hours; long half-life drugs: T1/2 > 8 hours.

7. Toxicity

Property	Value	Decision	Comment
hERG Blockers	0.668	●	■ Molecules with IC50 ≤10µM or ≥50% inhibition at 10 µM were classified as hERG+ (Category 1),<br>■ while molecules with IC50 >10µM or < 50% inhibition at 10µM were classified as hERG - (Category 0).<br>■ The output value is the probability of being hERG+, within the range of 0 to 1.
hERG Blockers (10um)	0.935	●	■ Molecules with IC50 ≤10 µM are classified as hERG+ (Category 1),<br>■ and molecules with IC50 > 10µM are classified as hERG- (Category 0).<br>■ The output value is the probability of being hERG+, within the range of 0 to 1.
DILI	0.349	●	■ Drug Induced Liver Injury.<br>■ Category 1: drugs with a high risk of DILI;<br>■ Category 0: drugs with no risk of DILI.<br>■ The output value is the probability of being toxic.
AMES Muta genicity	0.007	●	■ AMES Toxicity<br>■ Category 1: Ames positive(+);<br>■ Category 0: Ames negative(-);<br>■ The output value is the probability of being toxic.
Rat Oral Acute Toxicity	0.035	●	■ Rat Oral Acute Toxicity.<br>■ Category 0: low-toxicity, > 500 mg/kg;<br>■ Category 1: high-toxicity; < 500 mg/kg.<br>■ The output value is the probability of being toxic, within the range of 0 to 1.
FDAMDD	0.037	●	■ FDA Maximum (Recommended) Daily Dose.<br>■ Category 1: FDAMDD (+);<br>■ Category 0: FDAMDD (-);<br>The output value is the probability of being positive.
Skin Sensiti zation	0.63	●	■ Category 1: Sensitizer;<br>■ Category 0: Non-sensitizer.<br>■ The output value is the probability of being toxic, within the range of 0 to 1.
Carcinogeni city	0.677	●	■ Category 1: carcinogens;<br>■ Category 0: non-carcinogens;<br>■ The output value is the probability of being toxic.

ADMETlab 3.0

Eye Corrosion	0.587		■ Eye Corrosion<br>■ Category 1: corrosives;<br>Category 0: noncorrosives;<br>■ The output value is the probability of being corrosives.
Eye Irritation	0.996		■ Eye Irritation<br>■ Category 1: irritants;<br>Category 0: nonirritants;<br>■ The output value is the probability of being irritants.
Respiratory	0.139		■ Category 1: respiratory toxicants;<br>■ Category 0: non-respiratory toxicants.<br>■ The output value is the probability of being toxic, within the range of 0 to 1.
Human Hepatotoxicity	0.024		■ Human Hepatotoxicity<br>■ Category 1: H-HT positive(+);<br>■ Category 0: H-HT negative(-);<br>■ The output value is the probability of being toxic.
Drug-induced Nephrotoxicity	0.036		■ Category 0: non-nephrotoxic (-);<br>■ Category 1: nephrotoxic (+).<br>■ The output value is the probability of being nephrotoxic (+), within the range of 0 to 1.
Ototoxicity	0.051		■ Category 0: non-ototoxicity (-);<br>■ Category 1: ototoxicity (+).<br>■ The output value is the probability of being ototoxicity (+), within the range of 0 to 1.
Hematotoxicity	0.001		■ Category 0: non-hematotoxicity (-);<br>■ Category 1: hematotoxicity (+).<br>■ The output value is the probability of being hematotoxicity (+), within the range of 0 to 1.
Genotoxicity	0.0		■ Category 0: non-Genotoxicity (-);<br>■ Category 1: Genotoxicity (+).<br>■ The output value is the probability of being ototoxicity (+), within the range of 0 to 1.
RPMI-8226 Immunitoxicity	0.067		■ Category 0: non-cytotoxicity (-);<br>■ Category 1: cytotoxicity (+).<br>■ The output value is the probability of being ototoxicity (+), within the range of 0 to 1.
A549 Cytotoxicity	0.162		■ Category 0: non-cytotoxicity (-);<br>■ Category 1: cytotoxicity (+).<br>■ The output value is the probability of being ototoxicity (+), within the range of 0 to 1.
Hek293 Cytotoxicity	0.343		■ Category 0: non-cytotoxicity (-);<br>■ Category 1: cytotoxicity (+).<br>■ The output value is the probability of being ototoxicity (+), within the range of 0 to 1.
Drug-induced Neurotoxicity	0.055		■ Category 0: non-neurotoxic (-);<br>■ Category 1: neurotoxic (+).<br>■ The output value is the probability of being neurotoxic (+), within the range of 0 to 1.

8. Environmental toxicity

Property	Value	Comment
Bioconcentration Factors	0.201	■ Bioconcentration factors are used for considering secondary poisoning potential and assessing risks to human health via the food chain.<br>■ The unit is –log10[(mg/L)/(1000*MW)]
IGC_{50}	3.684	■ Tetrahymena pyriformis 50 percent growth inhibition concentration.<br>■ The unit is –log10[(mg/L)/(1000*MW)]
$LC_{50}FM$	1.448	■ 96-hour fathead minnow 50 percent lethal concentration.<br>■ The unit is –log10[(mg/L)/(1000*MW)]
$LC_{50}DM$	5.421	■ 48-hour daphnia magna 50 percent lethal concentration.<br>■ The unit is –log10[(mg/L)/(1000*MW)]

9. Tox21 pathway

Property	Value	Decision	Comment
NR-AhR	0.004	●	■ Aryl hydrocarbon receptor<br>■ Category 1: actives ;<br>■ Category 0: inactives;<br>■ The output value is the probability of being active.
NR-AR	0.002	●	■ Androgen receptor<br>■ Category 1: actives ;<br>■ Category 0: inactives;<br>■ The output value is the probability of being active.
NR-AR-LBD	0.001	●	■ Androgen receptor ligand-binding domain<br>■ Category 1: actives ;<br>■ Category 0: inactives;<br>■ The output value is the probability of being active.
NR-Aromatase	0.075	●	■ Category 1: actives ;<br>■ Category 0: inactives;<br>■ The output value is the probability of being active.
NR-ER	0.111	●	■ Estrogen receptor<br>■ Category 1: actives ;<br>■ Category 0: inactives;<br>■ The output value is the probability of being active.
NR-ER-LBD	0.049	●	■ Estrogen receptor ligand-binding domain<br>■ Category 1: actives ;<br>■ Category 0: inactives;<br>■ The output value is the probability of being active.
NR-PPAR-gamma	0.001	●	■ Peroxisome proliferator-activated receptor gamma<br>■ Category 1: actives ;<br>■ Category 0: inactives;<br>■ The output value is the probability of being active.
SR-ARE	0.006	●	■ Antioxidant response element<br>■ Category 1: actives ;<br>■ Category 0: inactives;<br>■ The output value is the probability of being active.

ADMETlab 3.0

SR-ATAD5	0.0	●	■ ATPase family AAA domain-containing protein 5<br>■ Category 1: actives ;<br>■ Category 0: inactives;<br>■ The output value is the probability of being active.
SR-HSE	0.464	●	■ Heat shock factor response element<br>■ Category 1: actives ;<br>■ Category 0: inactives;<br>■ The output value is the probability of being active.
SR-MMP	0.017	●	■ Mitochondrial membrane potential<br>■ Category 1: actives ;<br>■ Category 0: inactives;<br>■ The output value is the probability of being active.
SR-p53	0.064	●	■ p53, a tumor suppressor protein<br>■ Category 1: actives ;<br>■ Category 0: inactives;<br>■ The output value is the probability of being active.

10. Toxicophore Rules

**Property**	**Value**	**Comment**
Acute Toxicity Rule	0	■ 20 substructures;<br>■ acute toxicity during oral administration
Genotoxic Carcinogenicity Rule	0	■ 117 substructures;<br>■ carcinogenicity or mutagenicity
NonGenotoxic Carcinogenicity Rule	0	■ 23 substructures;<br>■ carcinogenicity through nongenotoxic mechanisms
Skin Sensitization Rule	0	■ 155 substructures;<br>■ skin irritation
Aquatic Toxicity Rule	0	■ 99 substructures;<br>■ toxicity to liquid(water)
NonBiodegradable Rule	0	■ 19 substructures;<br>■ non-biodegradable
SureChEMBL Rule	0	■ 164 substructures;<br>■ MedChem unfriendly status
FAF-Drugs4 Rule	0	154 toxic substructures from FAF-Drug4

ADMETlab 3.0
