# Supplementary material for: Integrative Network Toxicology Reveals Potential Molecular Targets Linking Plasticizer Exposure to Inflammatory Gastrointestinal Disorders
Source: Genes (Basel). 2026 Jun 7;17(6):667. doi: 10.3390/genes17060667 (PMC13299468; doi:10.3390/genes17060667)
Supplement: Supplementary file 1 [file genes-17-00667-s001.zip › Supplementary File S2.pdf]

Table S1. The structures of the compounds.

Structure	Compounds	SMILES sequences
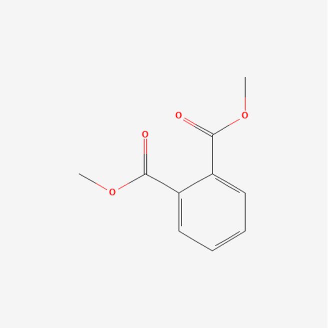	DMP	COC(=O)C1=CC=CC=C1C(=O)OC
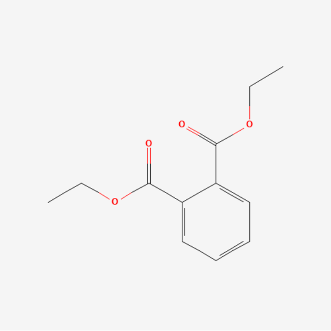	DEP	CCOC(=O)C1=CC=CC=C1C(=O)OCC
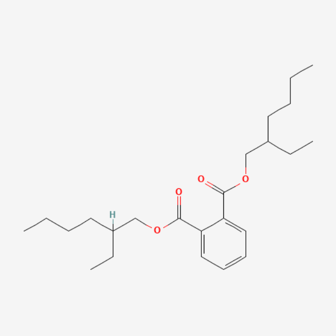	DOP	CCCCC(CC)COC(=O)C1=CC=CC=C1C(=O)OCC(CC)CCCC
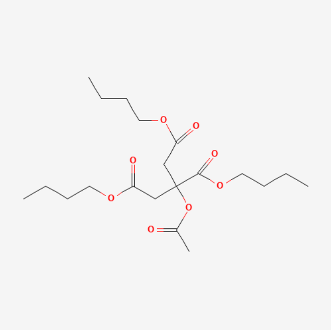	ATBC	CCCCOC(=O)CC(CC(=O)OCCCC)(C(=O)OCCCC)OC(=O)C

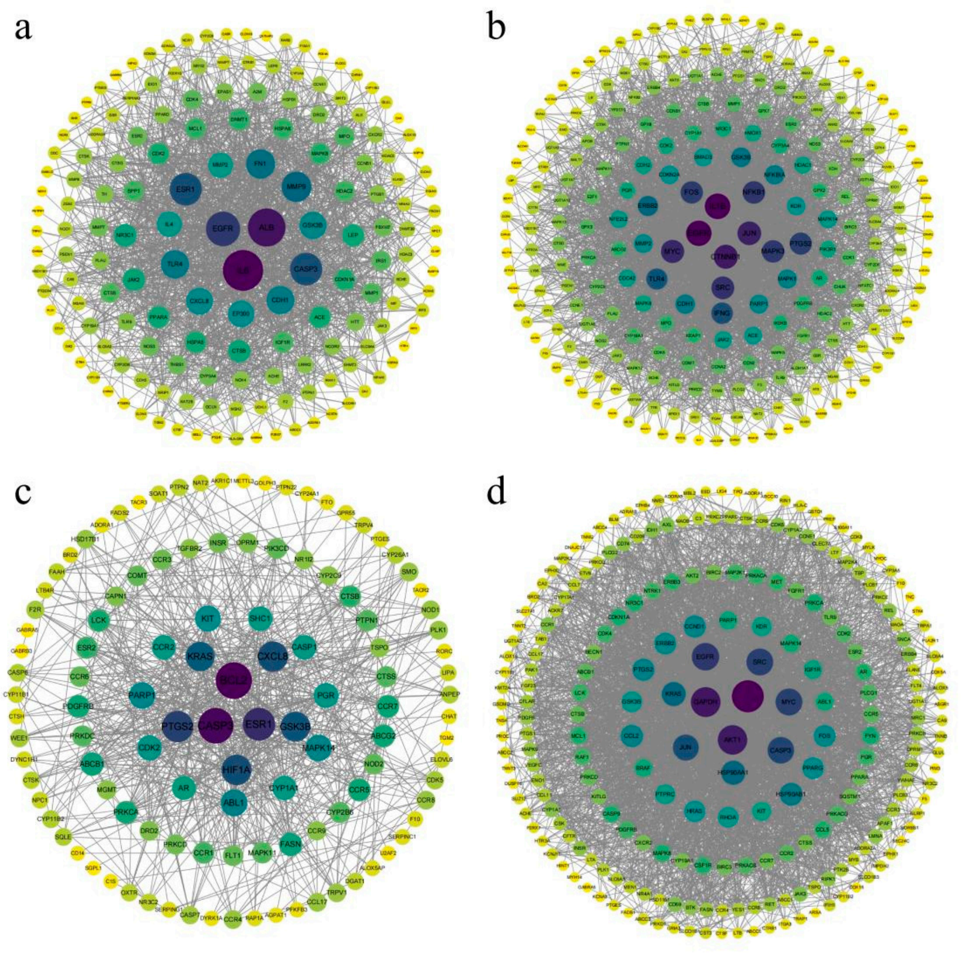

Figure S1. (a-d) PPI network of overlapping targets

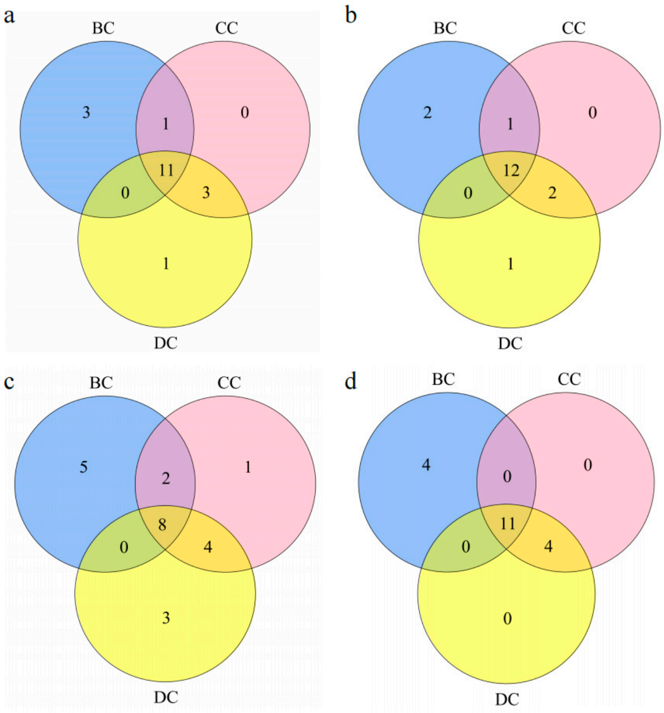

Figure S2. (a-d)Venn diagram showing the intersection of the top 15 targets selected using three local topology methods (DC, BC, CC)

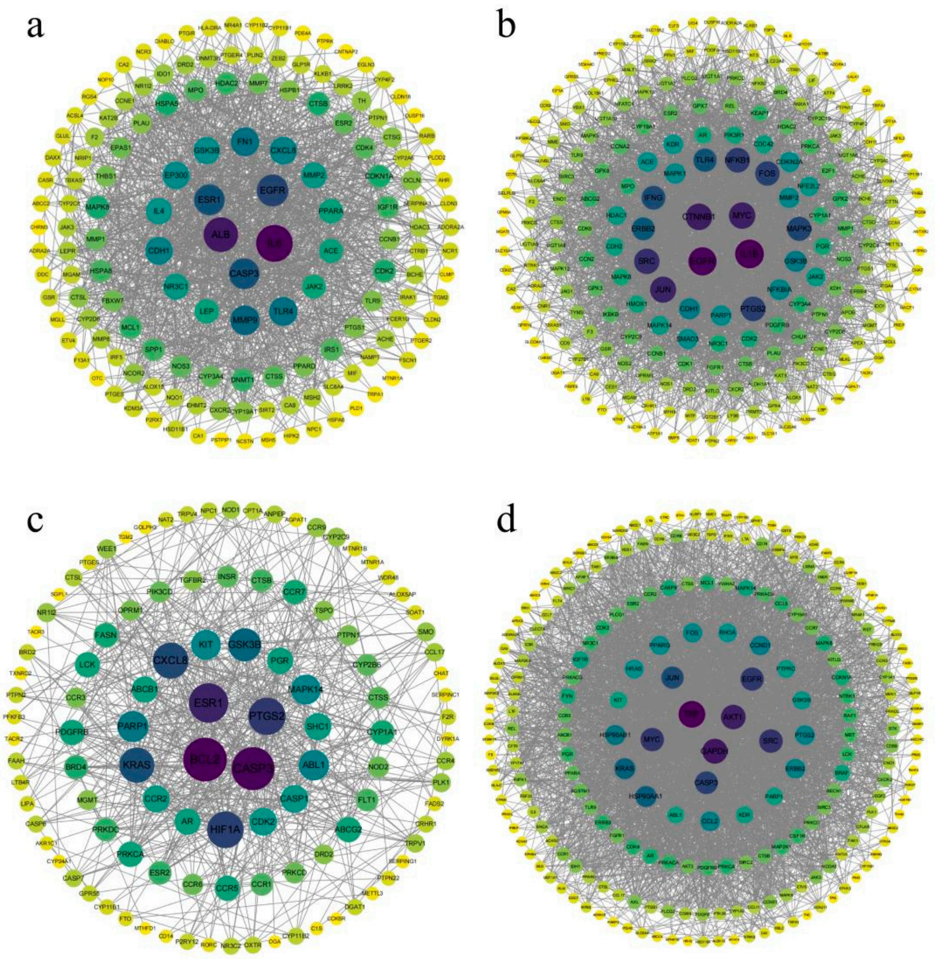

Figure S3. (a-d) PPI network of overlapping targets

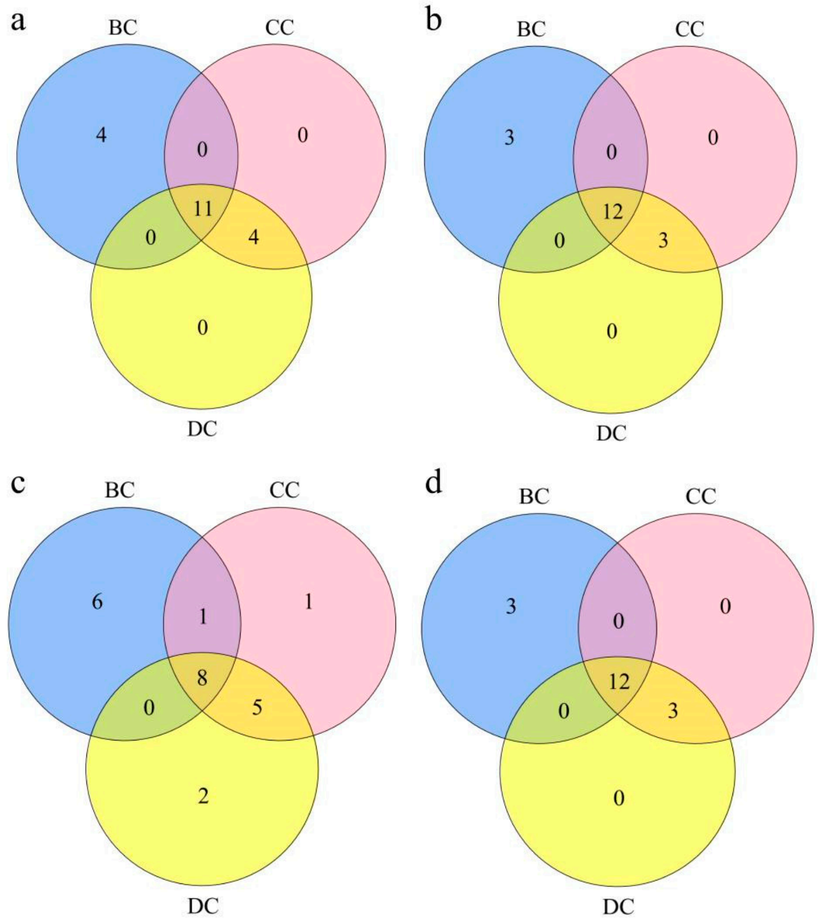

Figure S4. (a-d)Venn diagram showing the intersection of the top 15 targets selected using three local topology methods (DC, BC, CC)

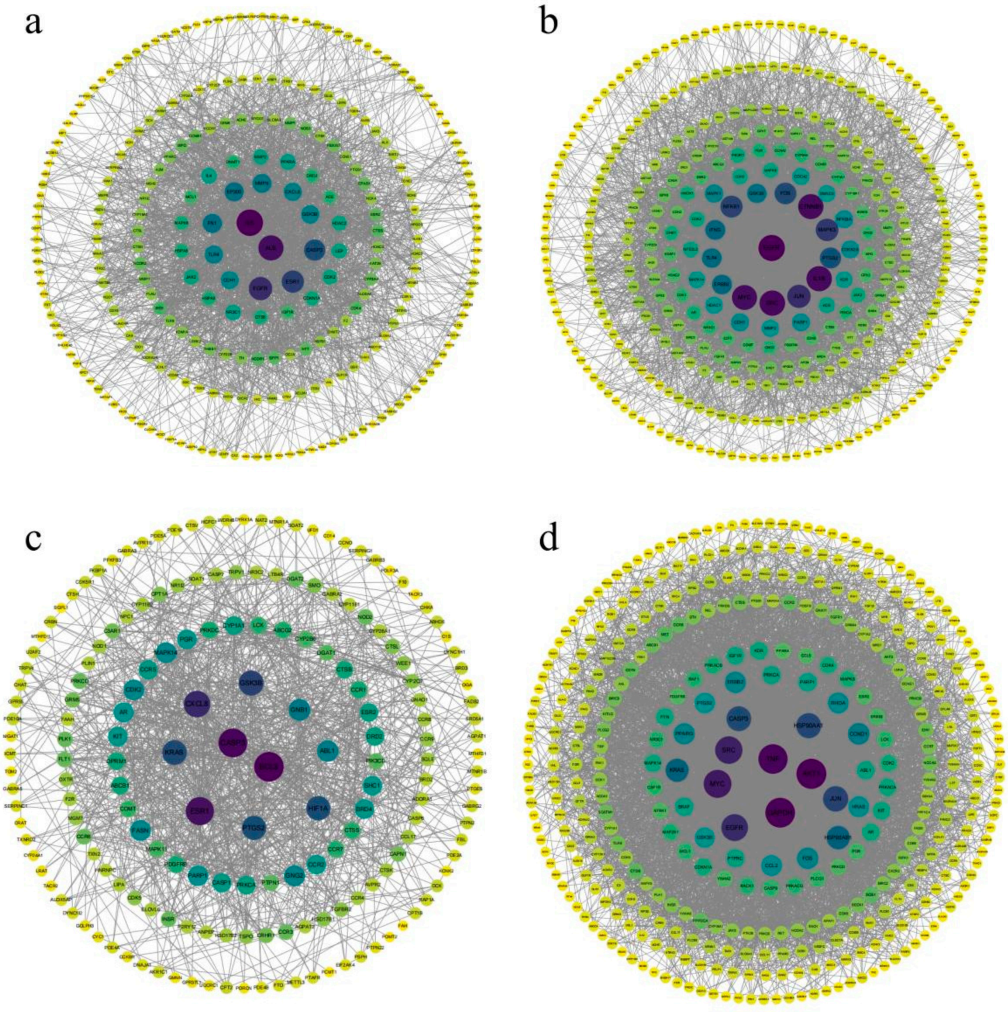

Figure S5. (a-d) PPI network of overlapping targets

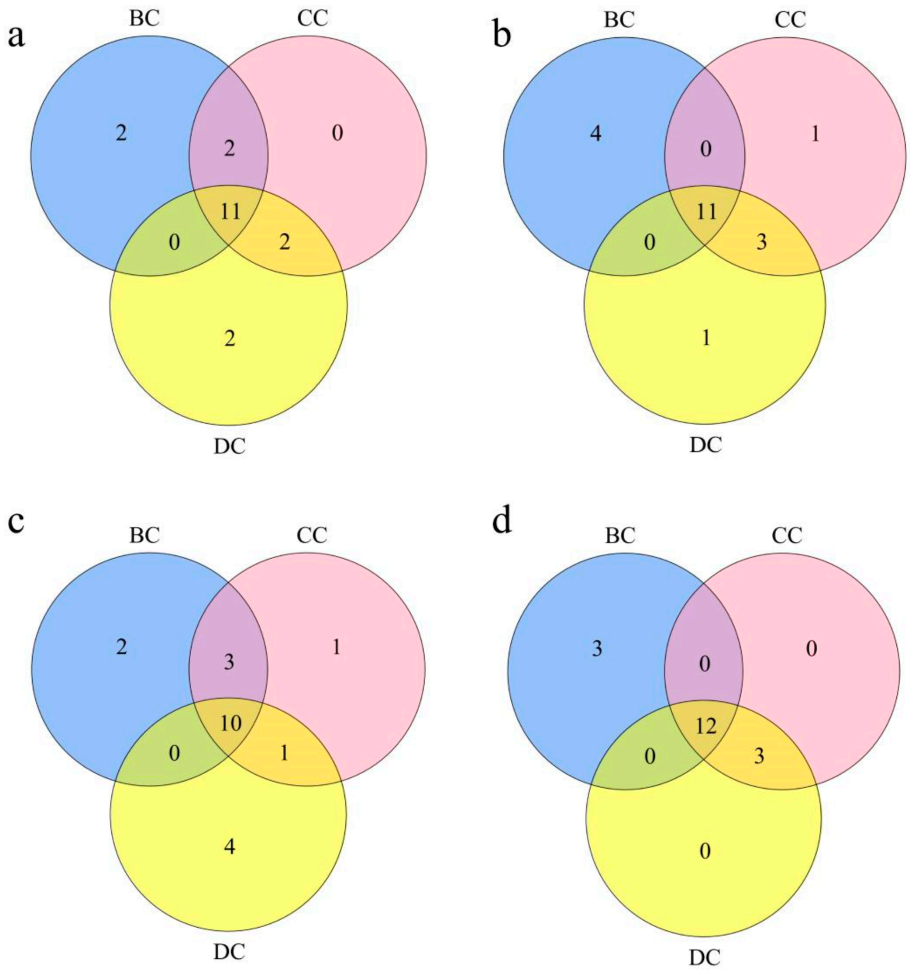

Figure S6. (a-d)Venn diagram showing the intersection of the top 15 targets selected using three local topology methods (DC, BC, CC)

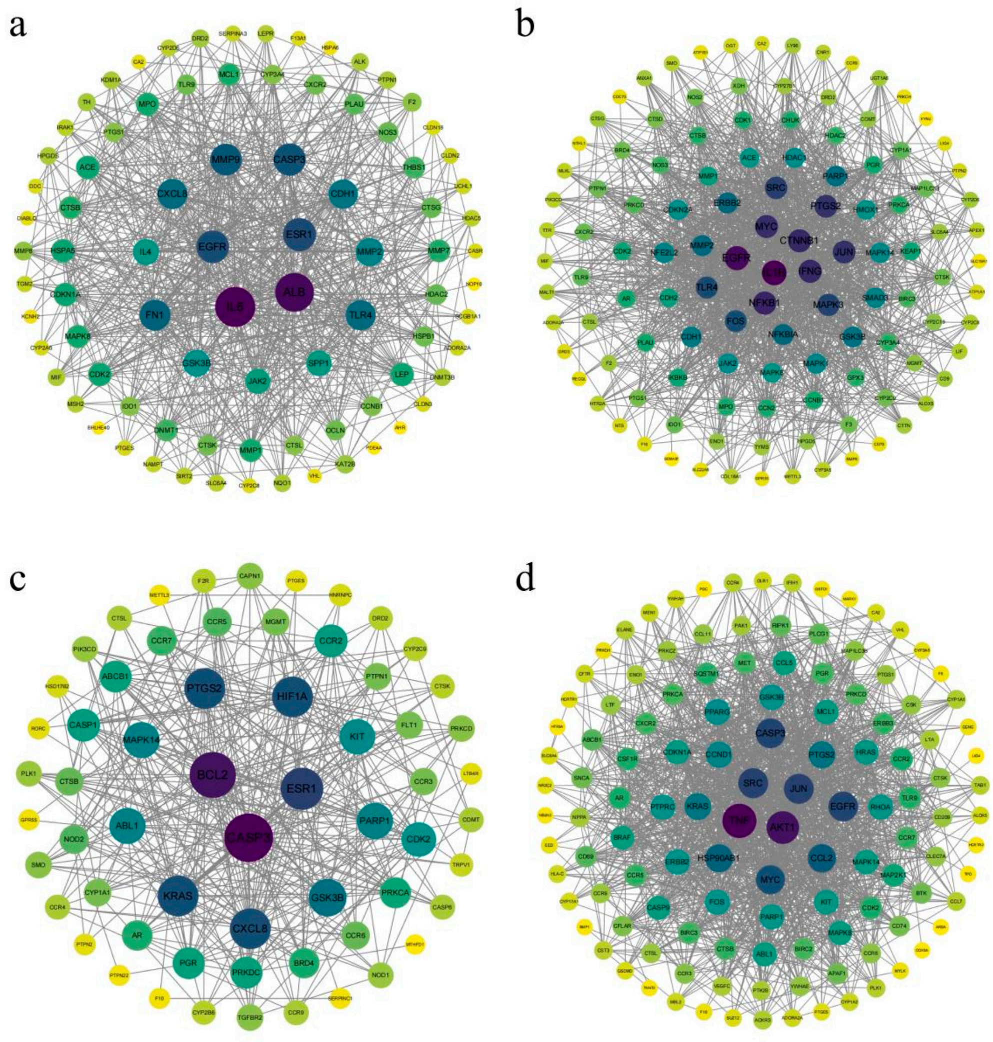

Figure S7. (a-d) PPI network of overlapping targets

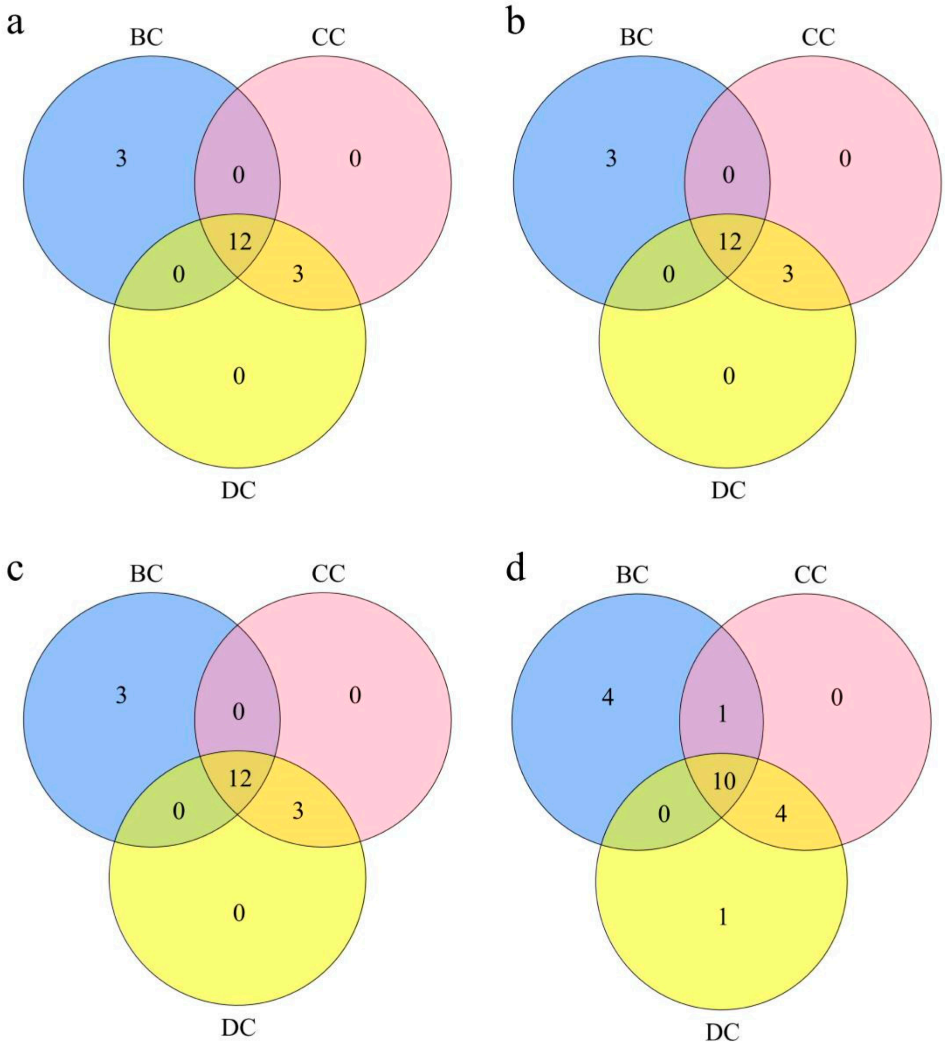

Figure S8. (a-d)Venn diagram showing the intersection of the top 15 targets selected using three local topology methods (DC, BC, CC)
